# OGG1 as an Epigenetic Reader Affects NFκB: What This Means for Cancer

**DOI:** 10.3390/cancers16010148

**Published:** 2023-12-28

**Authors:** Spiros Vlahopoulos, Lang Pan, Lokman Varisli, Garrett M. Dancik, Theodoros Karantanos, Istvan Boldogh

**Affiliations:** 1 First Department of Pediatrics, National and Kapodistrian University of Athens, Thivon & Levadeias 8, Goudi, 11527 Athens, Greece; 2 Department of Microbiology and Immunology, School of Medicine, University of Texas Medical Branch at Galveston, 301 University Blvd., Galveston, TX 77555, USA; lapan@utmb.edu; 3 Department of Molecular Biology and Genetics, Science Faculty, Dicle University, Diyarbakir 21280, Turkey; lokmanv@gmail.com; 4 Department of Computer Science, Eastern Connecticut State University, Willimantic, CT 06226, USA; dancikg@easternct.edu; 5 Division of Hematological Malignancies, Department of Oncology, Sidney Kimmel Comprehensive Cancer Center, Johns Hopkins University School of Medicine, Baltimore, MD 21218, USA; tkarant1@jhmi.edu

**Keywords:** OGG1, NFκB, gene regulation, microenvironment, EMT, innate immunity, cancer stem cells, lung cancer, acute myeloid leukemia, oxidant stress

## Abstract

**Simple Summary:**

OGG1-enabled NFκB activity can promote cancer progression via a number of key mechanisms that include phenotypic transitions of cancer and stromal cells, expression and secretion of molecules that modulate innate immunity, and alteration of the vascular network. Under oxidative stress, the supraphysiological levels of nongenotoxic 8-oxoGua, along with redox inactivation of OGG1 via cysteine oxidation and potential dimerization, provide multiple interfaces with redox-activated/modulated NFκB and other transacting factors, including Myc. This promotes not only cell proliferation, the expression of innate immune genes, and angiogenesis but also the metastatic capability of cells involving the expression of epithelial–mesenchymal transition genes. Mounting evidence supports the idea that increased levels of oxidative stress play a crucial role in the onset and progression of solid and lymphoid malignancies. This is achieved by gaining mutations and by activating signaling pathways that promote cell survival, cell proliferation, and resistance to treatment modalities.

**Abstract:**

8-oxoguanine glycosylase 1 (OGG1), which was initially identified as the enzyme that catalyzes the first step in the DNA base excision repair pathway, is now also recognized as a modulator of gene expression. What is important for cancer is that OGG1 acts as a modulator of NFκB-driven gene expression. Specifically, oxidant stress in the cell transiently halts enzymatic activity of substrate-bound OGG1. The stalled OGG1 facilitates DNA binding of transactivators, such as NFκB to their cognate sites, enabling the expression of cytokines and chemokines, with ensuing recruitment of inflammatory cells. Recently, we highlighted chief aspects of OGG1 involvement in regulation of gene expression, which hold significance in lung cancer development. However, OGG1 has also been implicated in the molecular underpinning of acute myeloid leukemia. This review analyzes and discusses how these cells adapt through redox-modulated intricate connections, via interaction of OGG1 with NFκB, which provides malignant cells with alternative molecular pathways to transform their microenvironment, enabling adjustment, promoting cell proliferation, metastasis, and evading killing by therapeutic agents.

## 1. Introduction

Cancer is driven by a set of drastic molecular changes that disrupt tissue homeostasis of a specific niche [1,2]. This disruption is rooted in changes in cellular chromatin that alter patterns of gene expression and thereby induce expression of molecules that facilitate cancer progression and interfere with the function of the immune system [3]. Disruption of tissue homeostasis redirects the normal succession of inflammation–regeneration into a process that perturbs immunity and enables growth of malignant cell clones with a tumor-initiating potential [4,5]. This affects tissue physiology.

Oxidative stress is a key factor that influences gene expression [6]. All types of antineoplastic treatment induce cellular stress aimed at killing tumor cells. However, cellular stress also triggers adaptation mechanisms that may enhance adaptability of malignant tumors. At least one of the adaptation mechanisms triggered by oxidative stress is the induction of inflammatory gene expression. Genes include cytokines, chemokines that mediate interactions between tumor and stroma, leading to cellular phenotypes that enable metastasis, protection of malignant cells from the immune system, and drug resistance.

Oxidant stress has been recognized as a key factor of immunosuppression, as both myeloid-derived suppressor cells and immunosuppressive extramedullary erythroid progenitor cells employ reactive oxygen species (ROS) to impair CD8+ T-cell responses against pathogens and tumors [7,8]. ROS promote cancer in diverse ways that affect multiple cell types that include malignant cells, as well as stromal and immune cells [9].

Here, we explore and discuss the intricate connections between redox imbalance, oxidative DNA base modifications, and the interactions of OGG1 with NFκB and Myc in the tumor microenvironment. Further research and understanding of the specific molecular mechanisms and their roles in tumor biology are essential for the development of targeted and effective therapeutic interventions.

## 2. Oxidative Stress Produces DNA Lesions, Turning OGG1 into an Epigenetic Reader

Nucleobases in DNA and RNA react differently with reactive entities, guanines (Gua) being the most sensitive due to their lowest oxidation potential among the five nucleobases. The interaction of Gua in RNA or DNA with reactive entities most often generates 7,8-dihydro-8-oxoguanine (8-oxoGua) and 2,6-diamino-4-hydroxy-5-formamidopyrimidine (FapyGua), which are the most abundant lesions in nucleic acids [10]. For 8-oxoGua, the anti-conformation is preferred in DNA; under replication or transcription (single stranded), it can take the syn conformation and pair with adenine (Hoogsteen base pairing). The 8-oxoGua: adenine base pair can produce a mutation in the following round of DNA replication, resulting in a G:C to T:A mutation [11].

8-oxoGua differs from guanine by only two atoms, but OGG1 can efficiently recognize it when paired with cytosine or 5-methylcytosine. 8-oxoGua is also recognized by human homologs of Nei-like glycosylases, primarily in single-stranded DNA [12,13,14]. After excision, the resulting apurinic/apyrimidinic (AP) sites are processed by AP endonuclease1/Ref1 (APE1/Ref1) or the polynucleotide kinase-phosphatase (PNKP) to form polymerase-ready 3′OH residues [15,16].

A question for these glycosylases is how they recognize modified bases in chromatin. Recent studies show, for example, that OGG1, a relatively low molecular weight polypeptide, searches for DNA base damage with the aid of its asymmetric surface charge distribution, requiring nearly no energy for migration (sliding) over the DNA duplex [17]. Substrate recognition and binding are accomplished via a complex set of molecular alterations, associating residues within the active site center with the oxidatively altered nucleotide to cleave the glycosyl bond. The removal of modified DNA base lesions ensures genome integrity and serves as a signal for cellular responses to oxidative stress [18]. It has been documented that oxidative stress induces the translocation of OGG1 (and other BER proteins) to open chromatin, which contains cis elements for transacting factors. These data imply that the searching activity of OGG1 for substrates is synchronized with chromatin remodelers that are transcriptionally active [19]. Cofactors such as histone marks, protein cofactors, or 8-oxoGua and FapyGua can increase the effective recruitment of OGG1 in chromatinized DNA [18,20,21].

Early studies documented that the OGG1 substrate, 8-oxoGua, is located within or in close proximity to cis elements for hypoxia-inducible factor-1 (HIF-1) within the promoter of vascular endothelial cell growth factor (VEGF), and it plays a crucial role in gene expression in hypoxic signaling [22]. Subsequent research identified 8-oxoGua as an epigenetic-like mark in the context of promoters within potential G-quadruplex, Z-DNA-forming and i-motif-forming sequences, as well as in promoters of proinflammatory genes and genes of high importance in epithelial-to-mesenchymal transitions [23,24,25,26]. These observations imply the site-specific generation of 8-oxoGua. Indeed, when Gua(s) are consecutively stacked, the oxidation potential of Gua is significantly lower and more prone to oxidation. This sequence arrangement stabilizes the cationic form of Gua, the precursor of 8-oxoGua. For example, in Gua quadruplexes, the oxidation potential of each Gua is lower by 0.1 V, making 5′-Gua extremely susceptible to oxidation. These regions are also called “hole domains” [27]. Such domains are often found adjacent to the transcription start site in the promoter region, 5′-UTR(s), and 3′ UTR(s) regions, providing evidence for the epigenetic role of generated 8-oxoGua [28]. In support, genome-wide mapping using quantitative 8-oxoGua immunoprecipitation-coupled sequencing showed the accumulation of 8-oxoGua, clearly correlating with the promoter, 5′-UTR, and occupancy of RNA polymerase II, as well as transcription [29], additionally, independent from Gua-rich sequences. However, studies have revealed a role for a nuclear flavoenzyme, lysine-specific demethylase 1 (LSD1), in mediating site-specific generation of ROS and consequently 8-oxoGua during demethylation of H3 lysine [30,31]. Thus, studies on LSD1 have provided an integrated mechanism by which 8-oxoGua is specifically generated at both enhancer and promoter sites. This, in turn, recruits OGG1 and DNA repair components, thereby activating estrogen-induced gene expression [31,32]. This is significant, as histone H3 Lys4 trimethylation sites are suggested to be oxidant-sensitive epigenetic marks that modulate gene expression under stress conditions [33,34]. Consequently, the distinction between modification(s) to DNA base(s), DNA repair, and epigenetics is now blurred, as studies have clearly identified changes in gene expression [35].

**Figure 1:**
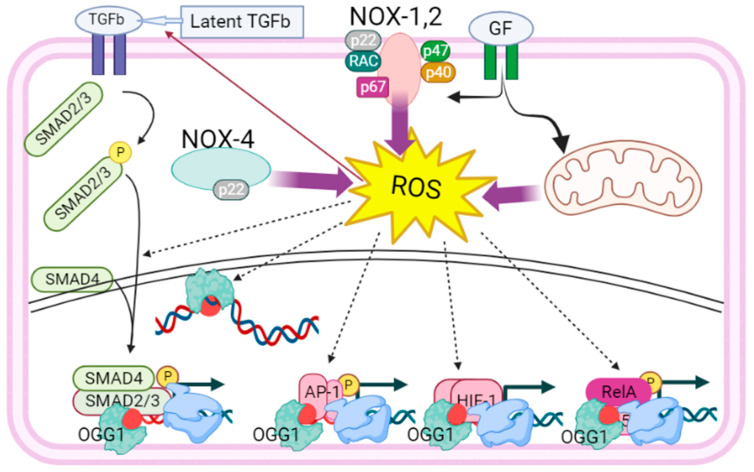
The proposed role of OGG1 in regulating gene expression related to tumor cell proliferation. In response to growth factors, hormones, metabolites, or environmental factors ROS are generated by NADPH oxidases and mitochondrial respiratory complexes. ROS signaling is crucial for activating growth factors (e.g., TGFβ1), transacting factors (e.g., NFκB, AP-1), and for modifying DNA bases (e.g., guanine → 8-oxoGua, an epigenetic mark). ROS also induce the oxidation of OGG1 at cysteine residues, inhibiting its glycosylase activity without affecting its ability to recognize/interact with intrahelical 8-oxoGua and the complementary cytosine.OGG1’s interaction with 8-oxoGua induces topographical rearrangements in DNA, resulting in an approximately 70-degree bending. This transient topographical change facilitates the DNA occupancy of transcription factors (e.g., SMADs, AP-1, HIF-1, NFκB) and components of the transcriptional machinery. Abbreviations: TGFβ—transforming growth factor beta; GF—growth factor; NOX—NADPH oxidoreductase; ROS—reactive oxygen species; SMAD—mothers against decapentaplegic homolog; AP-1—Jun activation-domain binding protein; HIF—hypoxia inducible factor; NFκB—nuclear factor kappa B. Created using Bio-Render: https://app.biorender.com (accessed on 9 September 2023).

The identification of 8-oxoGua as an epigenetic mark and the understanding of OGG1’s trans-regulatory function have been advanced by technologies used to map 8-oxoGua in the genome. These studies linked 8-oxoGua with the regulation of transcription, especially from enhancers, promoters, and untranslated regions (UTR), on a genome-wide scale [28,36,37,38] (Figure 1). High-throughput sequencing analysis found OGG1 in chromatinized DNA at 8-oxoGua over thousands of promoters, UTRs including genes implicated in innate immune responses (IIR, e.g., cytokines, chemokines, interleukins) and those responding to oxidative stress [39]. Further supporting the regulatory (epigenetic) role of 8-oxoGua in promoters, in-depth studies have demonstrated its key role during the transcriptional program of epithelial-to-mesenchymal transition (EMT) that is triggered by transforming growth factor β1 (TGFβ1) [40,41].

## 3. OGG1 Induces DNA Remodeling in Proximity of Transacting Factor’s Cis Elements

As detailed above, OGG1 scans DNA through diffusion, allowing it to search for modified Gua(s) (reviewed in [20]). Structural studies have shown that OGG1’s association with the epigenetic mark 8-oxoGua involves two major steps: (1) an initial enzyme–substrate interaction and (2) a fundamental remodeling of DNA [42], reviewed in [43]. During step 1, 8-oxoGua is extruded from the helix and placed into the OGG1 active-site pocket. In step 2, OGG1 interacting with the opposite cytosine causes it to become unstacked from adjacent bases, resulting in a sharp bending of the duplex DNA [42]. These slight but substantial changes in DNA structure around 8-oxoGua, in the context of chromatin, reduce the energy needed for transcription factors to occupy DNA and for the association of OGG1 with transacting factors and other proteins [13,44].

To better understand the role of OGG1 at 8-oxoGua in TFs DNA occupancy, it was shown that binding of NFκB homodimers (p50-p50) and heterodimers (p50-RelA/p65) to 8-oxoGua containing DNA is15- to 25-fold higher in the presence of OGG1 [35]. In the context of chromatin, OGG1 binding was mapped in close proximity to NFκB [39]. The binding of OGG1 to 8-oxoGua in promoters enhanced NFκB/RelA binding to cis-elements and facilitated the recruitment to the transcriptional complex of specificity protein 1 (SP1), transcription initiation factor II-D (TFIID), and RNA Pol-II. This led to increased expression of proinflammatory chemokines and cytokines in cultured cells and lungs of experimental animals [45].

Additionally, it was shown that DNA-bound OGG1 at 8-oxoGua interacted with mitogen-and stress-activated kinase 1, which phosphorylates NFκB/RelA at serine 276, inducing expression of innate cytokines and chemokines [46]. A recent study documented that OGG1 at 8-oxoGua in the context of chromatin recruited phosphorylated SMAD2/3 complex and NFκB to their cognate binding sequence located in promoters of tissue remodeling genes including alpha-smooth muscle actin (α-SMA), collagen (COL), and fibronectin (FN) for excessive gene expression [41,47]. Another recent study revealed that Myc oncogene binding to the E-box promoter recognition sequence required OGG1 under oxidative stress conditions [48]. Specifically, fluorescence polarization experiments discovered that the interaction with the Myc oncogene conformationally modified OGG1 at 8-oxoGua lesions and suspended OGG1′ glycosylase activity. In turn, OGG1 facilitated loading of Myc to E-box sequences [48]. The pathophysiological implication of OGG1 in promoting NFκB, SP1, TFIID, SMADs, Myc, as well as p-RNA Pol-II and transcription, for immune responses and Myc-induced tumor progression, holds unforeseen significance.

## 4. Direct Impact of OGG1-NFκB Interaction

OGG1 stimulates DNA binding of NFκB to several inflammatory gene promoters, thereby increasing expression of several cytokines, chemokines, and a number of other factors. This has been implicated in inflammatory syndromes in the lung and other organs [49,50]. Activated NFκB changes the protein occupancy and exposure of regulatory sequences on chromatin, enabling drastic changes in the phenotype of the cell [3]. In part, these changes occur by switching off numerous enhancer sequences and switching on other enhancers. This phenomenon is especially evident in extended enhancer sequences that are rich in transcription factor binding sites, and are termed stretch enhancers, or superenhancers [51,52]. Superenhancer changes are associated with cancer progression; in leukemia, specific feedforward loops are proposed to drive disease progression by switching on expression of NFκB downstream genes that include but are not limited to Myc [53,54,55]. After stimulation of embryonic kidney cells with TNFα, OGG1 and NFκB were found to bind to DNA recognition sites of organic cation transporter (OCT2), sex-determining region Y box transcription factor (SOX), and other families of regulators of cellular development by chromatin immunoprecipitation [56]. OGG1 and NFκB also activate RAS signaling, which is a known oncogenic driver [57,58].

Drastic changes in chromatin facilitate profound alterations in the cellular phenotype. While such events are crucial for the effective function of most cell types that are involved in inflammation, during cancer the induction of chromatin alterations enables cancer cells to acquire novel properties.

During innate inflammation, a sequential activation of different types of leukocytes takes place, induced by cytokines, chemokines, and adhesion factors. Malignant cells express subsets of genes that are normally induced in cell subtypes that are essential for survival of the organism [4]. As result, the sequence of events between inflammation and tissue regeneration is disrupted. Although cellular components of innate and adaptive immunity may be active, cancer cell niches are protected due to expression of immunosuppressive molecules in tumor nest [56].

## 5. Excessive Increase in ROS Sustains Inflammatory Gene Expression via OGG1 and NFκB

Despite evidence that in regulatory sequences of several genes OGG1-driven BER is essential for regulation of transcription, for inflammatory genes, excessive ROS was shown to allow for activation of transcription. Specifically, binding of enzymatically inactive OGG1 with the substrate is key to regulating oxidant stress-induced proinflammatory gene expression: an excision-deficient mutant (K249Q) was even a more potent activator of gene expression than wildtype OGG1, whereas mutant OGG1 with impaired substrate binding was not [59].

**Figure 2:**
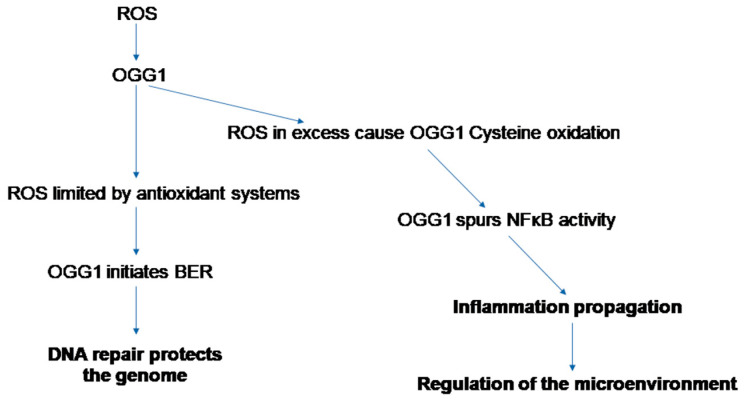
ROS that exceed an OGG1 variant-specific threshold halt OGG1 enzymatic activity temporarily to modulate the function of transcription factor NFκB. Despite evidence for transcription factor modulation by enzymatically active OGG1, here, we present the type of OGG1 reader activity that affects the microenvironment of a given cell through NFκB modulation. NFκB activates inflammation and this in turn can increase ROS levels. The positive transcription activation feedback loop generated in this manner can have a substantial impact in host tissue due to sustained expression of inflammatory mediators and disruption of physiological homeostatic function.

ROS cause oxidation of cysteine residues of OGG1, leading to a temporary halt in enzymatic activity [60]. However, this does not prevent transcriptional activity of the OGG1-associated NFκB to proceed [61] (Figure 2). Also, for Myc target genes, it was shown that OGG1–Myc interactions inhibit hOGG1 catalytic activity and recruit Myc to its promoters under oxidative stress [48]. Thus, there are multiple modes of OGG1 regulation of gene expression, as we noted earlier in detail in our study of ROS-generated epigenetic marks that are generated during respiratory viral infections [62].

## 6. A Convoluted Network Regulates NFκB Leading to a Complex Function in Cancer

NFκB is held inactive in the cytoplasm of most normal cells, bound by the polypeptide IκBα. Upon a cell receiving an inflammatory stimulus, IκBα is degraded and NFκB enters the nucleus to regulate gene expression (reviewed in [63]); the result of NFκB activation depends on the assortment of posttranslational modifications and interactions with other proteins, also including OGG1 [35]. Although the degradation of IκBα occurs at the proteasome in most cell types, it may be accomplished by other proteolytic systems, making pharmacological inhibition of the NFκB pathway in this step unsuccessful in some cases.

In particular, the lysosome and calpain may also degrade IκBα [64,65,66]. Especially in advanced cancer cells after exposure to chemotherapy, cell stress may activate biomolecule degradation at the lysosome to prevent cell death and provide essential metabolic intermediates. Most drugs cause cellular stress, and through the lysosome cell, stress itself becomes a driver for cancer progression [67].

NFκB activity in normal cells induces a number of negative feedback mechanisms that protect tissues from aberrant initiation of inflammatory cascades, which causes damage to functional tissue components though the immune system. However, in cancer cells, the network of feedback signal pathways to NFκB is not intact, leading, on the one hand, to protection of cancer cells from the immune system, and, on the other hand, to destruction of noncancerous tissue [3,4,56]. Recent data indicate that NFκB is often aberrantly active in malignant cells, and this activity can also be initiated or maintained by cell stress, enabling at least some tumor cell clones to survive under conditions that would be lethal to the original tumor.

In an impressive demonstration, restoring negative feedback to NFκB by a myeloid cell-targeted miR-146a mimic that prevented excessive NFκB activation in myeloid cells not only alleviated lethal inflammation in a chimeric antigen receptor (CAR) T-cell-induced cytokine release syndrome model of xenotransplanted B-cell lymphoma, but was also cytotoxic to leukemia cells in vitro and in vivo; it inhibited NFκB target genes expression and thereby thwarted progression of disseminated HL-60 promyelocytic leukemia [68]. Conversely, chronic activation of NFκB and expression of inflammatory genes in mouse hematopoietic stem cells leads to a “myeloid bias” during aging [69] and predisposes to leukemias [70].

In diverse types of cancer, constitutive NFκB activity enables malignant cells to survive oncogene activation, tumor suppressors, radiation, drug treatments, extensive genetic alterations, and the surveillance of both innate and adaptive immune cells [3,5]. However, more important is the fact that NFκB confers cancer cells flexibility in the capacity to acquire or relinquish stem cell attributes. Specifically, on the one hand, NFκB facilitates efficient induction of expression for antiapoptotic genes, and for the protooncogene encoding transactivator c-Myc, which prepares a cell for rapid growth by drastic changes in expression of metabolic enzymes [4]. On the other hand, NFκB confers to cancer cells the capacity to adopt stem cell features [71,72] by multiple indirect pathways that culminate in the acquisition of immortality and resistance to oxidative stress [3,73]. Malignant cells can thereby switch between different metabolic and phenotypic states.

## 7. Oxidative Stress as a Driver for Cancer Plasticity

Cancer progression succeeds after adaptation of malignant cells to the physiological, pathological, and pharmacological conditions of host tissue, which result from a combination of endocrine factors, drug treatment, and the resulting cellular stress: cancer cells that survive cytotoxic conditions show adaptation to cell stress [74]. Exposure of cells to increased oxidative stress provides a powerful stimulus to NFκB-driven gene regulation [75]. This is exemplified by NFκB interaction with OGG1 [35,47,76], which redirects NFκB activity and impairs immunity [76]. In contrast, enzymatically active OGG1 guards genome integrity through either lesion repair or elimination of cells with malignant potential, to maintain the homeostasis of the host, which might depend on the magnitude of guanine oxidation [77].

The Impact of OGG1 on cancer development can thus be dissociated from its function in DNA repair [56]. This is a fact that can be best illustrated by the OGG1 genetic allele variant that carries a C-to-G substitution at codon 326, giving rise to a protein product with a cysteine in place of serine in amino acid residue position 326. This OGG1 variant differs from the wildtype (wt) OGG1 in several aspects, including subcellular localization. OGG1 (S326C) is imported to the nucleus but is not found in nucleoli, in contrast to wtOGG1. The serine residue at position 326 is a potential phosphorylation site, and when OGG1 is phosphorylated at S326, it also exhibits nucleolar localization in addition to its association with soluble and condensed chromatin [78]. Due to the lack of this posttranslational modification, carriers of the OGG1 variant have impaired (lower) repair efficiency, especially in stress conditions, and are more susceptible to oxidation or nitrosation than those with wtOGG1 [79]. This allelic variant of OGG1 (S326C) is identified in over 50% and 40% of Asian and Caucasian populations, respectively [80].This naturally occurring polymorphic OGG1 variant has decreased activity, increased capacity to dimerize via Cys-Cys disulfide bond formation [81,82], it is more sensitive to inactivation by oxidative stress, and does not recover from oxidant stress after stimulation of cells with physiological concentration of TNFα [83] and therefore may provide a more effective enhancement of NFκB-driven gene expression. Specifically, the enrichment of OGG1 on chromatinized DNA, likely at its substrate for a prolonged period of time, is due to the loss of its enzymatic activity by ROS in cellulo, due to the lower threshold of OGG1 repair activity inhibition by oxidant stress.

Although cancer is a highly heterogeneous disease, at least two powerful examples demonstrate that OGG1 S326C can be implicated in malignant progression.

- Lung cancer: In advanced inoperable non-small cell lung cancer (NSCLC) patients who received treatment with platinum-based chemotherapy, the OGG1 genetic allele variant carrying a C-to-G substitution at codon 326 was associated with poor progression-free survival [84,85]. The imbalance between the S326C OGG1-enhanced inflammation and the delayed S326C OGG1 repair activity would be expected to impact lung pathology, especially in neoplastic tissue. Indeed, S326C Ogg1 was linked to shorter progression-free survival in inoperable NSCLC [84]. It should also be noted that the monoallelic Ser/Cys and biallelic Cys/Cys variants of OGG1 are potentially implicated in carcinogenesis, as they have previously been linked to increased susceptibility to various human malignancies including endometrial cancer [86], colorectal cancer [87], breast cancer [88], oropharyngeal squamous cell carcinoma [89], and prostate cancer [90,91].
- In acute myeloid leukemia (AML), OGG1 S326C was observed more frequently in patients experiencing relapse compared to other patients (28.9% vs. 8.9%, odds ratio = 4.10, 95% confidence interval = 1.35–12.70, *p* = 0.01), and those with the S326C variant exhibited a shorter relapse-free survival [92]. Remarkably, oxidative stress appears to have a key role in the outcomes of acute erythroid leukemia, which is a subtype of AML with particularly poor survival. This is suggested by the following facts:
  (a) In erythroleukemia cells (in vitro), the lipid peroxidation byproduct 4-hydroxynonenal, a reactive aldehyde [93], modulates Myc expression [94]. This provides a downstream target for stalled OGG1, with relevance for AML: increased oxidant stress stalls OGG1, which becomes an activator for NFκB-regulated gene expression; Myc is a known target gene of OGG1 [95]. Besides being a downstream NFκB target gene, Myc is a key regulator in malignant cells, and AML cells in particular [96,97,98]. In AML, NFκB transcriptional activity plays an established role in fostering tumor progression through paracrine secretion of cytokines in the microenvironment. However, the constitutive activation of NFκB, attributed to aberrant feedback, is associated with diverse advantages for AML cells, especially for leukemia stem cells [99,100,101]. This makes NFκB-activating modules, such as stalled, substrate-bound OGG1, a very strong candidate intervention target in AML.
  (b) The RNA expression of ALDH1A1, which encodes a key 4-hydroxynonenal (HNE) detoxifying enzyme, is increased in bone marrow samples from erythroleukemia patients [102]. Therefore, upstream of stalled OGG1, there are candidate targets for AML treatment too: the cause of OGG1 stalling, being oxidative stress, affects the intracellular milieu through lipid peroxidation products, namely reactive aldehydes. An enzyme that protects cells from reactive aldehydes is ALDH1A1. It is therefore not surprising that ALDH1A1 RNA is overexpressed in erythroleukemia and in AML poor prognosis patients in general.

What is important in terms of disease outcome is that AML patients of the poor prognosis risk group show frequent elevation of RNA expression for the enzymes ALDH1A1 and ALDH2, and this elevation is associated with an increased risk of death [102]. In the same study, ALDH1A1 encoding RNA was linked to an AML stemness signature. These findings are consistent with research that shows ALDH enzymatic activity protecting AML cells from chemotherapy and ALDH1A1 null AML cells susceptible to chemotherapy, while ALDH1A1 null patients belong to the favorable risk group [103,104]. Therefore, for patients, the capacity of AML cells to survive increases in reactive aldehydes is associated with a poor prognosis and disease progression, and can be traced, at least in part, to increased expression of ALDH enzymes [105,106,107,108].

In AML, there is a solid connection of NFκB with malignant progression, but nevertheless, interventions based on inhibiting NFκB or its associated mediators such as bromodomain and extra-terminal domain (BET) protein BRD4 [109] have not proven clinically effective [110,111]. This could be associated with the convoluted mode of NFκB regulation in cancer cells. This mode, however, exposes a number of potential intervention targets, both upstream and downstream from NFκB activation.

Leukemia stem cells exhibited resistance to BET inhibitors both ex vivo and in vivo, independently of Myc expression, and in connection with an upregulation of WNT/β-catenin signaling, which appeared essential to the manifestation of resistance [112]. Also, non-stem cells of leukemia could develop resistance to BET inhibition, yet this time, the resistance was linked to restoring Myc expression and required transcriptional plasticity. Chromatin immunoprecipitation-coupled sequencing and self-transcribing active regulatory region sequencing of enhancer profiles revealed that BET-resistant states are characterized by remodeled regulatory landscapes, involving the activation of a focal Myc enhancer that recruits WNT machinery in response to BET inhibition [113].

Thus, depending on the leukemia cell type, both BRD4 and Myc can become dispensable under certain conditions.

## 8. A Model for Malignant Progression for Cancers Driven by Changes in Oxidative Stress Response of OGG1

A key aim in research for cancer treatment is to target mechanisms that permit malignant cells to withstand cytotoxic chemotherapy. One general aspect of cancer cell resistance mechanisms to chemotherapy is the development of clones with increased capacity to respond to cellular stress. Chemotherapy is administered to kill cancer cells and proves especially effective in killing cancer cells that operate error-prone systems of biomolecule synthesis and processing. However, exposure of cancer cells to chemotherapy or any other cytotoxic conditions tends to select for clones that operate efficient cell stress adaptation mechanisms, which often act by inducing the removal of mediators of cell death, or by preventing accumulation of cytotoxic metabolites. The latter is particularly important for leukemia cells exposed to chemotherapy [73,114].

Therefore, we may work with a model for malignant progression for cancers driven by changes in oxidative stress response. Specifically, increased oxidative stress initially inactivates several enzymes by the formation of adducts with lipid peroxidation products such as HNE [115]; one such enzyme is OGG1, which during increased oxidative stress becomes a transcriptional coactivator for transactivators such as NFκB [35], enhancing expression of several inflammatory genes, including cytokines that enable transformation of the tumor microenvironment, and other proteins that protect cancer cells from the immune system, despite activation of several types of immune cells [56]. While a number of tumor cell clones may die, activation of biomolecule recycling by lysosomes can rescue malignant cells that adapt to increased oxidative stress. Enhanced activity of enzymes such as ALDH1A1, which detoxifies reactive aldehydes, and of enzymes that participate in glutathione reduction allows for adapted cancer cells to escape apoptosis, necrosis, ferroptosis, and cuproptosis [73].

Cellular stress itself thereby becomes a driver of tumor cell escape from destruction.

In the example of AML progression, when viewed from the angle of ALDH enzymes, a refinement of the model is possible: ALDH1A1 is crucial in specific AML stages. It coincides with specific challenges to a cell’s malignant function and survival. ALDH2 is not stage specific. In general, ALDH2 overexpression coincides with a bad outcome in AML. ALDH2 functions more efficiently to detoxify mitochondrial acetaldehyde, which is rather constitutively produced. This means that specific stages of AML, which can be viewed as distinct phases when a given leukemic cell phenotype dominates, need increased ALDH1A1 activity. HNE can offer a plausible explanation for this exclusivity of specific AML stages for the hazard of ALDH1A1 overexpression:

(a) Proliferating leukemic blast cells have high metabolic activity and Myc expression, and may tolerate higher ROS [116], with accompanied high levels of HNE. These cells are resistant to lysosomal inhibition because they are not dependent on the recycling of biomolecules. OGG1 enzymatic function is disabled by HNE [115], and OGG1 transcriptional activity via recruited transcription factors such as NFκB delivers high expression of inflammatory mediators, feedforward ROS-activated oncogenes [56,57,117,118], as well as immunosuppressive molecules that, in cooperation with increased oxidant stress in the microenvironment, inhibit the function of effector T-cells. HNE also keeps ALDH2 activity low by forming adducts with ALDH2 [119], which allows for increased generation of mitochondrial acetaldehyde. However, as increased ROS damage chromatin [120], these cells may generate increased genetic diversity for AML, even if a large portion of AML cell clones undergoes cell death. To note: studies have revealed that the DNA occupancy of Myc to its E-box in the chromatin is facilitated by OGG1.In oxidative stress conditions present in AML cells, OGG1 undergoes dimerization via cys28 residues. However, it can still recognize 8-oxoGua, and through its interaction with Myc, it promotes binding at 8-oxoGua in the promoter. Additionally, data indicate that Myc significantly decreases the glycosylase activity of OGG1, resulting in a prolonged binding to the epigenetic mark. Myc recruitment to E-box DNA motifs by OGG1 in oxidizing cellular milieu can lead to inflammatory conditions and to enhanced gene expression of Myc target genes [48].
(b) Latent leukemic stem cells have low metabolic activity, tolerate low ROS levels, and keep limited levels of HNE, which is readily detoxified by enzymes such as ALDH1A1 and other systems, such as glutathione-based enzymes [121,122]. These cells are dependent on lysosomal activity and are therefore sensitive to lysosomal inhibition [123]. OGG1 enzymatic function in these cells is within optimum range [92,124] and protects from some chemotherapy agents such as cytarabine [125], and ALDH2 activity detoxifies mitochondrial acetaldehyde, further protecting cellular macromolecules. Latent cells offer a low profile to the immune system, probably by expression of “don’t eat me” signals and suppression of key antigen exposure [126,127]. Excessive increase in HNE abrogates the colony-forming capacity of AML cells [128]. Increased expression of enzymes such as ALDH1A1, which form a first-line defense from HNE, protects cells from chemotherapy-triggered lipid peroxidation and enables the emergence of chemoresistant AML [73]. ALDH-high leukemia cells are also associated with a poor patient prognosis [129].

It is important to note that the latent leukemic cells described in (b) are not propagated as a fixed cellular phenotype. These cells rather represent a key phenotypic intermediate, which easily converts into proliferating blast cells when exposed to excessive cell stress: the presence of a substantial antioxidant capacity simply offers to leukemia cell clones the critical opportunity to grow long enough to generate new resistant subclones, which under hostile conditions may become the dominant cellular phenotype. It is therefore important to bear this in mind when adjusting treatment design.

This distinction between cell states should become evident experimentally. Specifically, when research models are monitored for a time window that is long enough to allow for the development of phenomena of cellular evolution, some form of equilibrium between quiescent and blast cells may become temporarily established. The cell subclones observed experimentally may acquire properties of subclones that develop during leukemia progression in a living mammalian organism.

**Figure 3:**
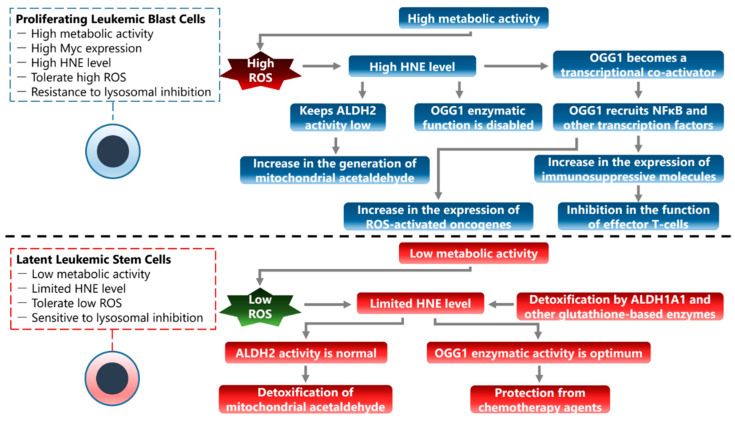
A proposed model for two basic phenotypes of AML stem-like cells that may also coexist in a form of dynamic equilibrium that is regulated by the metabolic state of their microenvironment.

Such phenomena should be anticipated or expected to also occur in the organism of a patient that experiences leukemia relapse, or leukemia that is refractory to treatment (Figure 3). This conceptual model may provide an explanation on why the ROS/Myc/BRD4 axis is not indispensable for leukemia: while Myc overexpression and activation “burns the brakes” of tissue homeostasis and creates the conditions that enable Myc-driven carcinogenesis, excessive cellular stress and ROS enable alternative transcriptional programs that allow for development of leukemic stem cell clones. It would not be surprising if such observations also emerge for solid tumors with respect to transcriptional flexibility and the growth in resistance to anticancer treatment.

## 9. Phenotypic Transitions in the Microenvironment

In addition to its effects on cancer and stromal cells, oxidative stress can modulate endothelial cell function, which is suggested by research on the OGG1-NFκB interaction in chromatin [62]. Dysfunction of the vascular endothelial barrier in cancer has been implicated in disease pathology, progression, and outcome [6,130], as vascular endothelial cells have a key role during tumor metastasis [131], and the interaction between cancer cell and endothelium has been documented in research models for migration through the blood–brain barrier [132]. In particular, endothelial–mesenchymal transition has been proposed to occur after anthracycline treatment and to initiate vascular remodeling [133]; anthracyclines are known triggers of NFκB activation [134] and specifically initiate endothelial-to-mesenchymal transition [135] on the one hand, and on the other hand, NFκB activation protects cancer cells from apoptosis [136].

However, endothelial dysfunction in cancer also has severe symptoms that may prove that endothelial injury is lethal on its own [137]. It is critical to restore endothelial function in cancer by controlling oxidative stress, as an optimum level of endothelial redox normalization is required to allow for extravasation of effector T-cells into the tumor microenvironment [138].

Not only too high but also too low oxidative stress may prove critically detrimental in cancer: cyclic adenosine monophosphate (cAMP) activators by inducing oxidative stress impaired glioma-derived endothelial cell differentiation in vivo, normalized the tumor vessels, and altered the tumor immune profile, especially increasing the influx and function of CD8+ effector T-cells [139].

In a study of broader scope, the OGG1 enzymatic activity was examined as a target for cancer cell killing due to the proven dependence of several cancer cell types on PARP and BER. Indeed, it was shown that several types of cultured cancer cells can be killed at micromolar concentrations of OGG1 inhibitors. Moreover, an acute lymphoblastic leukemia cell line needed OGG1 activity to grow both in vitro and in vivo [140].

Lastly, OGG1 is a mediator of pulmonary fibrosis sparked by bleomycin; the underlying mechanism is alveolar epithelial–mesenchymal transition (EMT) and can be inhibited by small-molecule OGG1 inhibitor TH5487 or enhanced by OGG1 overexpression [141,142]. EMT is a process involved in lung fibrosis and cancer metastasis associated with NFκB and SMAD activation [143,144]. It was shown that NFκB activation and EMT were essential for the expression of immunosuppressive molecule PD-L1 by TNFα plus TGFβ1-stimulated A549 lung adenocarcinoma cells [145]. Specifically, TGFβ1 decreased the expression of DNA methyltransferase DNMT1 and that resulted in PD-L1 promoter demethylation whereas TNFα induced the NFκB pathway that promoted expression of the demethylated PD-L1 promoter.

In a previous study, platelet-derived TGFβ activated the Smad and NFκB pathways in cancer cells, resulting in EMT and enhanced metastasis in vivo; inhibition of NFκB signaling in cancer cells or ablation of TGFβ1 expression in platelets protected against lung metastasis in vivo [146,147]. A model for cancer metastasis proposes that it is initiated by EMT that is possibly spurred by TGFβ1-expressing macrophages, and in the destination niche, it is accomplished by a mesenchymal-to-epithelial transition that is facilitated by resident cells such as Ly6C+ myeloid progenitor, which have been shown to express extracellular matrix proteoglycan versican in metastatic lungs [148]. What is important is that inhibiting OGG1 suppressed the numbers of M2macrophages [142,149], which is a phenotype shown to promote angiogenesis [150] and immunosuppression [151].

## 10. Conclusions

These findings suggest that OGG1-enabled NFκB activity can promote cancer progression via a number of key mechanisms that include phenotypic transitions of cancer and stromal cells, expression and secretion of molecules that modulate innate immunity, and alteration of the vascular network. Mounting evidence supports the idea that increased levels of oxidative stress play a crucial role in the onset and progression of solid and lymphoid malignancies. This is achieved by inducing genomic instability, promoting cell survival, signaling for cell proliferation, and increasing resistance to treatment modalities (reviewed in [152]). Multiple mechanisms have been described for aberrant ROS generation in malignant cells, particularly of myeloid origin. This could involve dysfunction of the mitochondrial respiratory chain, activation/dysregulation of various oxidoreductases/antioxidant machineries, and/or changes in metabolism, allowing for cell survival and proliferation under constant oxidative stress. Under oxidative stress, the supraphysiological levels of nongenotoxic 8-oxoGua, along with redox inactivation of OGG1 via cysteine oxidation and/or dimerization, provide multiple interfaces with redox-activated/modulated NFκB and other transacting factors, including Myc. This promotes not only cell proliferation, the expression of innate immune genes, and angiogenesis but also the metastatic capability of cells involving the expression of epithelial–mesenchymal transition (EMT) genes. Taken together, facilitation by OGG1 of NFκB and Myc binding to their cognate motifs on DNA can alter the course of malignancy and render cancer cells resistant to chemo/radiotherapy. Therefore, we speculate that small molecules that inhibit generation of the epigenetic-like mark 8-oxoGua, and/or recognition of its genomic substrate by OGG1, may have clinical utility, as this was suggested in pioneering work [125]. A deeper understanding of the interactions between ROS, OGG1, NFκB, and Myc could help in understanding and ultimately treating malignant disease.
